# Headache during Childhood

**Published:** 1892-10

**Authors:** 


### HEADACHE DURING CHILDHOOD.

Seven groups of headache may be classified:

1. Headache from rapid growth. It is usually frontal, is increased by exercise, and co-exists with pain in the joints, periostoses, and hypertrophy of the heart. Treatment: muscular repose, tonics, liberal diet, phosphate of lime, malt beer

2. Headache from intellectual activity. It occurs in intelligent and excitable children, who study too much; or in backward children, who acquire their lessons with difficulty.

Treatment: for the first class of cases, cessation of intellectual work, physical exercise, but not so severe as to produce fatigue, luke-warm baths. In the second class of cases, the work may be continued in moderation, plenty of exercise being enjoined.

3. Headache from digestive troubles. It occurs in children who eat too much or too fast, and occurs in one to three hours after eating. Treatment: properly regulated hygiene and diet; by bitter tonics before eating, warm drinks after eating. Constipation should be overcome.

4. Headache of nervous origin. It occurs in children who are excited by their manner of living. It is premonitory of future neuropathies, epilepsy, and hysteria. Treatment: baths, walking, massage, valerian, aconite, and antipyrin for the hysterical; belladonna and bromides for the epileptics. They should avoid taking cold.

5. Headache in children of gouty or rheumatic diathesis. It is sometimes accompanied by intense congestive phenomena, which simulate meningitis. There are manifestations of hereditary antecedents; there are neuralgias, arthralgias, myalgias; the urine contains phosphates, oxalates, and urates. Treatment: moderate diet, exercise in the open air, vapor baths with friction, laxatives, alkalines, salicylate of soda in doses of from twenty-five to thirty centigrammes, and tincture of colchicum in ten to fifteen drop doses daily.

6. Headache from anæmia and poisoning. In the first case it is due to bad air and hygiene, in the second to malaria, carbonic oxide, to excessive medication, to uræmia. Treatment: it should vary with the cause.

7. Headache from injury to the sensory organs. There may be chronic conjunctivitis, or keratitis, or iritis, which should be treated locally, and also by the internal use of sulphate of quinine in large doses. Troubles of refraction, hypermetropia, and astigmatism must be treated with suitable glasses. There may be mucous polypi in the nose, or hypertrophies, which call for local treatment. There may be adenoid vegetations in the ears, otitis, or foreign bodies in the auditory canal, which call for suitable treatment.—*Archives of Pediatrics*; *Abstract and Index.*

